# AQP4-IgG and mood disorders: Case series of neuromyelitis optica spectrum disorder

**DOI:** 10.1016/j.bbih.2026.101177

**Published:** 2026-01-12

**Authors:** Qing Xu, Yanlin Han, Shuzhan Gao, Siyi Wang, Yanyan Lu, Kuan-Pin Su, Xijia Xu

**Affiliations:** a Department of Psychiatry, The Affiliated Brain Hospital of Nanjing Medical University, Nanjing, 210029, Jiangsu, China; b Department of Psychiatry, Nanjing Brain Hospital, Clinical Teaching Hospital of Medical School, Nanjing University, Nanjing, 210029, Jiangsu, China; c Office of Research and Development, Asia University, Taichung, Taiwan; d An-Nan Hospital, China Medical University, Tainan, Taiwan; e College of Medicine, China Medical University, Taichung, Taiwan

**Keywords:** Mood disorders, AQP4-IgG, NMOSD, Brain, Bipolar disorder, Major depressive disorder

## Abstract

Neuromyelitis optica spectrum disorders (NMOSD) are autoimmune inflammatory demyelinating conditions primarily affecting the optic nerves and spinal cord. While NMOSD pathogenesis is mediated by aquaporin-4 antibody (AQP4-IgG) autoimmunity, increasing evidence suggests significant comorbidity with affective symptoms. The neuropathological mechanisms underlying this comorbidity, however, remain incompletely understood. We reported two cases of female patients with concurrent NMOSD and mood disorders that illustrate a shared neuroinflammatory etiology. Case 1, a 26-year-old female, presented with a decade-long history of mood instability, diagnosed as bipolar disorder (BD) mixed episode, which coincided with her NMOSD diagnosis. Case 2, a 25-year-old female, initially presented with major depressive disorder (MDD) but was subsequently diagnosed with NMOSD following the acute onset of neurological symptoms. Both patients were, serum AQP4-IgG positive and exhibited demyelinating changes in the frontal lobe. Following immunosuppressive and psychiatric treatment, both patients experienced marked improvement in neurological function, suicidality, and affective symptoms. These case reports suggest a bidirectional relationship between NMOSD and mood disorders, likely mediated by AQP4-IgG-driven neuroinflammatory responses. AQP4-IgG screening may serve as a critical tool to distinguish organic mood disorder from primary psychiatric conditions, particularly in patients with atypical neurological symptoms.

## 1 Introduction

Neuromyelitis optica spectrum disorders (NMOSD) refer to a severe autoimmune disease affecting the central nervous system, characterized by inflammatory demyelination, and the aquaporin-4 antibodies (AQP4-IgG) serve as a specific biomaker for NMOSD (Jarius et al., 2020). The pathogenesis of NMOSD involves the binding of AQP4-IgG to aquaporin-4 (AQP4) expressed on astrocytes, which initiates a cascade of pro-inflammatory cytokines and chemokines, activates inflammatory cells, and ultimately leads to oligodendrocyte injury. This process contributes to blood-brain barrier disruption and central nervous system demyelination (Jasiak-Zatonska et al., 2016), resulting in clinical manifestations such as optic neuritis, longitudinally extensive myelitis, and posterior horn syndrome.

Bipolar disorder (BD) and major depressive disorder (MDD), collectively referred to as mood disorders, are highly comorbid conditions associated with substantial functional impairment and an increased risk of mortality (Solmi, M. et al., 2022). Patients with autoimmune diseases like NMOSD face a significantly higher risk of depressive and anxiety symptoms than the general population (Tan et al., 2024). Approximately 58 % of patients with NMOSD experience affective dysregulation, however, only 40 % receive appropriate treatment, and half of those treated individuals show a response (Chavarro et al., 2016). Chronic pain and long-term disability associated with NMOSD are key contributors to depressive symptoms, anxiety symptoms, and insomnia. However, comorbid psychiatric and neurological conditions remain undertreated, and limited attention has been directed toward the shared neuropathological mechanisms underlying these disorders, underscoring the need for enhanced clinical awareness.

Here, we report two cases of female patients with NMOSD who exhibited prominent affective symptoms, including depressive and manic features occurring at distinct stages of their disease course. Notably, the observed neuroimaging abnormalities, together with the improvement of affective symptoms following immunotherapy, suggest a potential association among AQP4-IgG-mediated autoimmunity, demyelination-like central nervous system lesions, and affective dysregulation.

## 2 Case report

### 2.1 Case 1

A 26-year-old female was admitted to the psychiatric ward on March 30, 2025, with a chief complaint of recurrent mood instability over the past 10 years and increased irritability over the preceding two weeks.

**Fig. 1:**
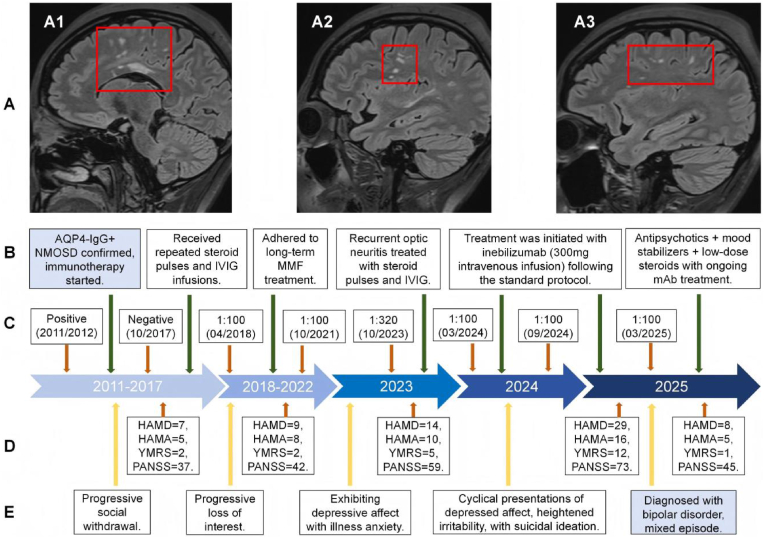
Clinical and Biological Features in Case 1. **A** T2WI features: demyelinating lesions predominantly involve the corona radiata (A1), frontal lobe, and centrum semiovale (A2, A3); **B** Neurological treatment timeline; **C** AQP4-IgG titer timeline; **D** Psychiatric assessment scale; **E** Recurrent episodes of emotional instability and irritability. HAMD: Hamilton Depression Rating Scale; HAMA: Hamilton Anxiety Rating Scale; IVIG: Intravenous Immunoglobulin; mAb: Monoclonal Antibody; MMF: Mycophenolate Mofetil; PANSS: Positive and Negative Syndrome Scale; T2WI: T2-Weighted Imaging; YMRS: Young Mania Rating Scale.

The patient experienced progressive visual loss in 2011. Positive serum AQP4-IgG results were detected, leading to a diagnosis of NMOSD. Following initiation of glucocorticoid (GC) therapy, partial clinical improvement was observed. No depressive or manic episodes were reported at the time of the initial NMOSD onset. Several years after the initial NMOSD diagnosis and in the absence of overt neurological relapse, the patient gradually developed depressive symptoms in 2015, including persistently low mood, diminished interest and motivation, reduced energy, and decreased activity levels. Between 2015 and 2023, she reported frequent mood instability, with intermittent fluctuations in irritability, sleep, and energy. In November 2023, the patient experienced a recurrence of visual deterioration and was treated with methylprednisolone pulse therapy combined with intravenous immunoglobulin, followed by a gradual tapering of corticosteroid dosage. Mild irritability and sleep disturbance were noted during the tapering phase but did not meet criteria for mood disorders. In February 2024, maintenance therapy with inebilizumab was initiated (Fig. 1B). Throughout the treatment period, serial assessments of AQP4-IgG levels were conducted (Fig. 1C).

Beginning on March 15, 2025, two weeks preceding hospitalization, she experienced manic symptoms characterized by noticeable irritability, aggressive verbal expression and physically violent behavior. Upon admission, in addition to these above manic symptoms, she exhibited profound anhedonia, impaired social functioning, persistent pessimism, recurrent suicidal behaviors, and physical aggression toward her parents. Additionally, she harbored delusional beliefs that her illness was caused by the malicious intentions of others. Based on the longitudinal course of depressive and manic episodes and the clinical presentation, a diagnosis of BD was established in accordance with the 11th revision of the International Classification of Diseases (ICD-11) diagnostic criteria (Fig. 1E).

According to the evoked potential report, myelin damage resulted in bilateral dysfunction of the visual conduction pathways. On May 2, 2025, brain magnetic resonance imaging (MRI) showed multiple white matter hyperintensities in the frontal lobes, bilateral corona radiata, and centrum semiovale, consistent with demyelinating pathology (Fig. 1A1-A3), which showed minimal change compared to the findings observed during her NMOSD relapse in November 2023.

During hospitalization, treatment was initiated with intramuscular haloperidol to manage acute agitation, followed by gradual titration of aripiprazole to 10 mg/day and lamotrigine to 50 mg/day for the management of manic and depressive symptoms. Concurrently, she is still taking a low dose of GC. Following treatment, the patient's mood stabilized, with no recurrence of vision loss or other NMOSD related neurological symptoms was observed.

### 2.2 Case 2

A 25-year-old female was admitted to the psychiatric ward on April 10, 2025, with a chief complaint of depressive symptoms persisting for one year, characterized by recurrent low mood, and acute bilateral visual blurring that had emerged over the prior week.

In April 2024, the patient initially developed depressive symptoms characterized by persistent low mood and marked anhedonia, without neurological complaints. By October 2024, these symptoms had progressed into a clinically significant depressive episode, accompanied by substantial occupational impairment, ultimately leading to job resignation and subsequent social withdrawal. In December 2024, she experienced epigastric discomfort and recurrent postprandial vomiting. Clinical deterioration became evident in March 2025, with the onset of psychomotor retardation, severe social isolation, persistent bilateral visual loss, and suicidal ideation, prompting psychiatric admission. A diagnosis of MDD was established according to ICD-11.

During hospitalization, she was treated with fluvoxamine titrated to 50 mg/day, olanzapine up to 2.5 mg/day, and lorazepam up to 1 mg/day. However, she gradually developed numbness and weakness in the left arm and shoulder, accompanied by progressively increasing difficulty in lifting the left upper limb. Brain MRI revealed signal abnormalities in the medulla oblongata, prompting transfer to the neurology department for further evaluation and management.

**Fig. 2:**
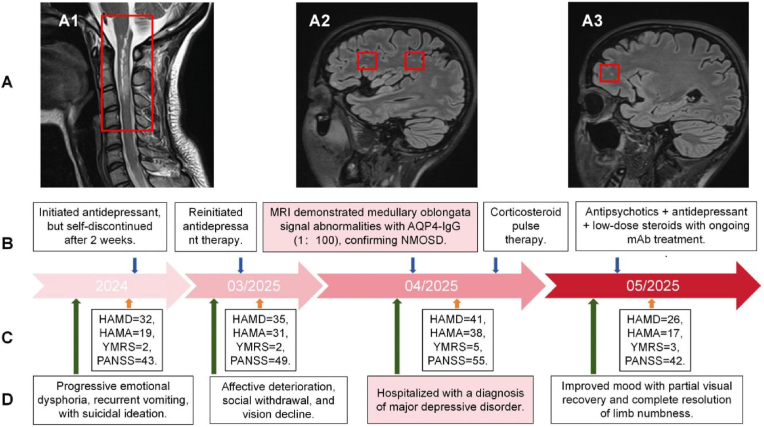
Clinical and Biological Features in Case 2. **A** T2WI features: longitudinally extensive transverse myelitis lesion (A1), demyelinating lesions predominantly involve the frontal lobe and centrum semiovale (A2,A3); **B** Diagnostic and therapeutic timeline; **C** Psychiatric assessment scale; **D** Symptom progression timeline. HAMD: Hamilton Depression Rating Scale; HAMA: Hamilton Anxiety Rating Scale; mAb: Monoclonal Antibody; PANSS: Positive and Negative Syndrome Scale; T2WI: T2-Weighted Imaging; YMRS: Young Mania Rating Scale.

In the neurology department, screening for central nervous system demyelinating antibodies was performed, revealing positive AQP4-IgG serology, which established the diagnosis of NMOSD. MRI demonstrated abnormal signals in the optic chiasm, midbrain, cerebral peduncles, medulla oblongata, and upper cervical spinal cord, along with demyelinating lesions in the frontal and parietal lobes (Fig. 2A). The patient received methylprednisolone pulse therapy followed by a gradual taper and underwent six sessions of double-filtration plasmapheresis. Subsequently, her visual function improved, enabling her to perceive the outlines of people. Numbness and weakness in the left limb were alleviated compared to baseline. Maintenance immunotherapy with satralizumab was then initiated (Fig. 2B). As NMOSD-directed treatment progressed, the patient's depressive symptoms concurrently improved. During the treatment, fluvoxamine was subsequently switched to sertraline due to concerns regarding tolerability and its higher potential for cytochrome P450-mediated drug interactions. Sertraline was considered more suitable for continued treatment given its favorable safety profile, fewer pharmacokinetic interactions, and broader clinical experience supporting its use in patients requiring long-term antidepressant therapy. Thereafter, sertraline was titrated to 50 mg/day to further alleviate residual depressive symptoms, olanzapine was maintained at 2.5 mg/day to control agitation, and alprazolam was added up to 0.4mg/day as a short-term anxiolytic for insomnia relief.

## 3 Discussion

We report two female patients with co-occurring NMOSD and mood disorders. Both were positive for AQP4-IgG, one meeting diagnostic criteria for BD and the other for MDD. Both presented with suicidal ideation and demyelination-like abnormalities in the frontal lobes. Following combined immunomodulatory and psychotropic treatment, both patients demonstrated significant improvement in neurological function and affective symptoms.

In addition to high expression in the optic nerve and spinal cord, AQP4 is broadly distributed throughout multiple regions of the central nervous system (Wingerchuk and Lucchinetti, C. F., 2022). AQP4-IgG-mediated neuroinflammation may contribute to dysfunction within the brain's emotional regulation network, representing a neurobiological substrate rather than merely a psychological reaction to chronic illness. Postmortem studies show AQP4 expression in both cortical and subcortical white matter of the frontal lobe, with higher levels in cortical layers than in subcortical regions (Adiele et al., 2025). Neuropsychological evidence consistently underscores the pivotal role of the frontal lobes, particularly the prefrontal cortex, as central to emotional processing and affective regulation (Aron et al., 2014). In our two AQP4-IgG-positive patients, neuroimaging findings indicated structural abnormalities in key frontal regions, including the prefrontal cortex, which may constitute a neural correlate of the affective symptoms observed in NMOSD.

BD and MDD are well-established psychiatric conditions with biological basis, manifesting as dysfunction in specific brain regions and their interconnected neural circuits. Current neurobiological models suggest that patients with affective symptoms exhibit impaired prefrontal regulation of limbic circuits involved in emotion processing (Jabbi et al., 2020). Within this circuit, the anterior cingulate cortex has been shown to correlate with the severity of suicidal ideation (Zhao et al., 2025). T2-weighted imaging (T2WI) is highly sensitive to increased tissue water content following myelin damage, making T2WI hyperintensities a widely accepted radiological indicator of potential demyelination. These hyperintensities are frequently observed in patients with mood disorders, and white matter hyperintensities have been independently associated with suicidal tendencies, suggesting a potential neurobiological correlate of suicidal behavior (Grangeon et al., 2010). Given the elevated suicide risk inherent among patients with mood disorders, the current paucity of epidemiological data on suicide in NMOSD patients represents a significant clinical gap. The two female patients presented herein exhibit structural abnormalities in the prefrontal lobe, which may contribute to cognitive dysfunctiona core symptom domain of mood disorders. The synergistic interaction between these cognitive deficits and affective dysregulation may increase vulnerability to suicidal ideation and behavior.

Many patients with autoimmune diseases like NMOSD cannot avoid using GC. GC exposure impairs GC receptor-mediated negative feedback of the hypothalamic-pituitary-adrenal (HPA) axis and induces structural alterations in the brain, thereby contributing to anxiety, depressive symptoms, memory deficits, and cognitive dysfunction (Wang, H et al., 2024). In both cases, several clinical features argue against GC-induced affective symptoms. The severity and duration of mood and behavioral changes were disproportionate to the overall treatment duration and dosage of GCs. Moreover, the temporal relationship between GC exposure and the onset of affective symptoms was inconsistent with typical GC-induced psychiatric syndromes. In Case 1, manic symptoms emerged during a low-dose maintenance phase rather than during high-dose initiation or rapid dose escalation, and in Case 2, depressive symptoms developed before the initiation of GC therapy. Taken together, these observations suggest that the affective symptoms cannot be attributed solely to GC use.

Our case reports suggest that when affective symptoms occur in the context of NMOSD, first-line treatment strategies should prioritize etiology-targeted immunotherapy rather than psychiatric medications alone. During the maintenance phase, proactive screening and early intervention for affective dysregulation are essential components of comprehensive disease management. Furthermore, our findings underscore the importance of considering AQP4-IgG testing and comprehensive MRI evaluation in patients with MDD or BD who present with mild neurological symptoms in psychiatric settings, such as vomiting or visual impairment.

We propose that comorbid NMOSD and mood disorders share a neuropathological basis linked to AQP4-IgG-mediated cascades. Postmortem studies have demonstrated that AQP4-IgG is associated with brain regions involved in mood regulation (Bernard, R. et al., 2011), suggesting a possible role in affective circuitry dysfunction. Beyond its diagnostic utility in NMOSD, AQP4-IgG may act as a causal pathogenic factor contributing to the development of BD or MDD, thereby representing a potential biological marker for autoimmune-related affective disturbances. Demyelination-like structural changes may constitute a core pathological mechanism connecting autoimmune neuroinjury with affective dysregulation. In both presented cases, serology confirmed AQP4-IgG positivity, and neuroimaging revealed demyelination-like abnormalities in the frontal lobes-key regions for emotion and cognition. Clinically, both patients exhibited significant mood disturbances and prominent suicidal ideation. Combination immunotherapy and symptom-targeted psychiatric treatment produced marked improvement in neurological and affective outcomes. Notably, neither patient had a family history of psychiatric disorders, suggesting that the observed phenotypes may arise primarily from neuroinflammatory processes rather than genetic predisposition. These converging clinical, serological, and neuroimaging findings collectively strengthen the plausibility of the proposed hypothesis.

However, these reports have several important limitations. These include a small sample size, the inability to establish causality based on clinical observations alone, the presence of multiple confounding factors, and the subjectivity of assessment methods. Therefore, this pathophysiological model remains hypothetical. Future studies should incorporate larger, well-characterized cohorts to validate the epidemiological profile of these comorbid conditions and to conduct longitudinal assessments of the association between microstructural alterations in the frontal-limbic system and serial measurements from mood and anxiety rating scales in patients with NMOSD.

## 4 Conclusion

AQP4-IgG positivity may be associated with affective symptoms in NMOSD patients. These case reports suggest a bidirectional association between NMOSD and mood disorders, likely mediated by AQP4-IgG-driven neuroinflammation. AQP4-IgG may serve as a key biomarker to differentiate secondary mood disturbances in individuals with underlying neurological pathology, although this remains a hypothesis requiring validation through larger, longitudinal studies. These cases underscore the need for heightened diagnostic vigilance in psychiatric practice to enable timely, mechanism-based interventions.

## CRediT authorship contribution statement

**Qing Xu:** Writing – review & editing, Writing – original draft, Visualization, Resources, Investigation, Data curation, Conceptualization. **Yanlin Han:** Writing – review & editing, Resources, Investigation, Conceptualization. **Shuzhan Gao:** Writing – review & editing, Resources, Investigation. **Siyi Wang:** Writing – review & editing, Investigation. **Yanyan Lu:** Writing – review & editing, Resources, Investigation. **Kuan-Pin Su:** Writing – review & editing, Visualization, Supervision, Formal analysis, Conceptualization. **Xijia Xu:** Writing – review & editing, Writing – original draft, Visualization, Supervision, Conceptualization.

## Informed consent

Written informed consent was obtained from the patient.

## Declaration of competing interest

The authors declare that they have no known competing financial interests or personal relationships that could have appeared to influence the work reported in this paper.

## Data Availability

Data will be made available on request.
